# Glucose variability increases during minimally invasive procedures in very preterm infants

**DOI:** 10.1007/s00431-022-04641-2

**Published:** 2022-10-06

**Authors:** Alfonso Galderisi, Giulia Res, Silvia Guiducci, Federica Savio, Sabrina Brigadoi, Laura Forlani, Biancamaria Mastrandrea, Laura Moschino, Elisabetta Lolli, Elena Priante, Daniele Trevisanuto, Eugenio Baraldi

**Affiliations:** 1 Department of Women’s and Children’s Health, Neonatal Intensive Care Unit, University of Padua School of Medicine, Via N. Giustiniani 3, 35128 Padova, Italy; 2 Institute of Pediatric Research, Padova, Italy; 3 Hopital Necker-Enfants Malades, Paris, France; 4 Department of Developmental and Social Psychology, University of Padua, Padova, Italy

**Keywords:** Neonatal glucose, Prematurity, Glucose variability, Nursing procedures, Continuous glucose monitoring, Preterm infants

## Abstract

The objective of this study is to assess the effect of neonatal procedures on glucose variability in very preterm infants. Preterm infants (≤ 32 weeks gestation and/or birthweight ≤ 1500 g) were started on continuous glucose monitoring (CGM) on day 2 of birth and monitored for 5 days. Minimally invasive (heel stick, venipunctures) and non-invasive (nappy change, parental presence) procedures were recorded. CGM data were analyzed 30 min before and after each procedure. The primary outcome was the coefficient of glucose variation (*CV* = *SD*/mean) before and after the procedure; SD and median glucose were also evaluated. We analyzed 496 procedures in 22 neonates (GA 30.5 weeks [29–31]; birthweight 1300 g [950–1476]). Median glucose did not change before and after each procedure, while CV and SD increased after heel prick (*p* = 0.017 and 0.030), venipuncture (*p* = 0.010 and 0.030), and nappy change (*p* < 0.001 and < 0.001), in the absence of a difference during parental presence.

*Conclusions*: Non-invasive and minimally invasive procedures increase glucose variability in the absence of changes of mean glucose.

**Table:** 

**What is Known:** *• Minimally invasive procedures - including nappy change - may increase neonatal stress in preterm infants*.	
**What is New:** *• Continuous glucose monitoring provides a quantitative measure of neonatal stress during neonatal care procedures demonstrating an increase of glucose variability.*

**What is Known:**

*• Minimally invasive procedures - including nappy change - may increase neonatal stress in preterm infants*.

**What is New:**

*• Continuous glucose monitoring provides a quantitative measure of neonatal stress during neonatal care procedures demonstrating an increase of glucose variability.*

## Introduction

Preterm infants experience multiple daily invasive and minimally invasive procedures during their care in the neonatal intensive care unit (NICU). Invasive and minimally invasive procedures, such as skin-breaking maneuvers, are managed by providing pharmacological and non-pharmacological analgesia [1–3].

The safety of continuous glucose monitoring (CGM) in preterm neonates has been largely explored, as well as the association of hyperglycemia with clinically relevant adverse neurological outcomes [4–10], thus suggesting a new tool to continuously monitor glucose changes in this population and the effect of neonatal care on glucose fluctuation, as a surrogate marker of neonatal stress.

Herein, we investigated the effect of nursing care procedures (nappy change), parental presence, and skin-breaking procedures (heel prick and venipuncture) on mean glucose and glucose variability in very preterm neonates by the use of CGM.

## Methods

### Population

We conducted a secondary analysis on a cohort of 22 very preterm infants enrolled in a randomized controlled trial at the University of Padua (NCT04347590).

Eligible subjects were preterm infants born at ≤ 32 weeks of gestation or with birth weight ≤ 1500 g admitted to the NICU of the University Hospital of Padua (Italy), within 48 h of birth. Babies with congenital malformations, known as chromosomal abnormalities or birth weight < 500 g, were excluded.

### Study procedures

CGM (Medtronic Enlite) was placed on the lateral side of the thigh preceded by adequate containment and analgesia with the use of pacifier and 0.3 ml 24% sucrose 2 min before the procedure. The device was calibrated as per manufacturer instructions.

### Data recording

The nursing staff recorded the timing of two groups of procedures: nursing care procedures (nappy change, parents’ presence) and skin-breaking procedures (heel stick, vascular access placement, or venous withdrawal from an existing access) for the 5 days following sensor positioning.

### Glucose outcome measures

**Fig. 1:**
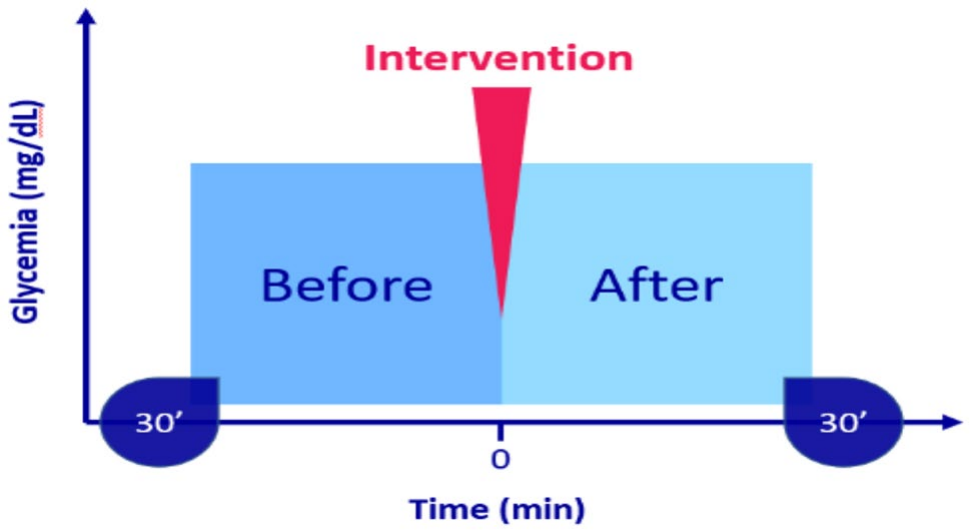
Study design

CGM data during the 30 min before the intervention and 30 min after the prespecified intervention (Fig. 1) were analyzed. Glucose variability was computed as coefficient of variation (*CV* = glucose standard deviation / mean glucose (mg/dL)).

### Statistics

Continuous variables were expressed as median, interquartile range (25th, 75th) if not normally distributed, or mean ± standard deviation; categorical variables were expressed as number (%)*.* For not normally distributed continuous variables, the Wilcoxon test was used to compare continuous variables. The difference of glucose measures before and after the procedures was quantified as percentage glucose change and computed as difference of mean glucose after and before the procedure over baseline glucose.

A linear mixed model analysis was conducted to compare the change of glucose profile before and after the procedures, with gestational age and birthweight accounted as covariates, after testing for collinearity.

Data were analyzed with STATA/SE 13.1 (StataCorp, Lakeway, TX, USA). Graphs were elaborated with Prism 8 (GraphPad Software).

## Results

Twenty-two neonates were enrolled, with a median gestational age of 30.5 weeks (29–32) and a median birth weight of 1300 g (950–1476), eight females (36%) and seven twins (32%). Gestational diabetes was recorded in five mothers (23%). Participants’ characteristics are detailed in Table 1.

**Table 1:** Baseline characteristics (*n* = 22)

Neonates	
Gestational age (wk)	30.5 (29–31.75)
Birthweight (g)	1300 (950–1476.25)
Small for gestational age, *n* (%)	5 (22.7)
Apgar 1’	6 (6, 7)
Apgar 5’	8 (8, 8)
Twins, *n* (%)	7 (32)
Sex, *n* (%) LOS, *n* (%) CRIB II	8 females (36.4) 7 (32%) 2 (1–3)
EI, *n* (%)	2 (9.1)
NEC, *n* (%)	5 (22.7)
Maternal characteristics and delivery	
Cesarean delivery, *n* (%)	17 (77.3)
Maternal diabetes, *n* (%)	5 (22.7)
PPROM, *n* (%)	5 (22.7)

Data are expressed as median (25th, 75th centile) or number (percentage)

*LOS* late onset sepsis, *CRIB II* clinical risk index for babies, *EI* endotracheal intubation, *NEC* nectrotizing enterocolitis, *PPROM* preterm premature rupture of membranes

We analyzed 496 procedures (343 nappy changes, 25 parental presence, 104 heel sticks, and 14 venipunctures).

During the monitoring period, we recorded 1.87% data loss of 35.9 h CGM monitoring (2154 min), out of a total of 1918.55 h of monitoring (115,113 min). The median loss of monitoring time for each baby was 1.4 h [IQR 0.8–2.0] / 83.4 h of CGM use [IQR 47.4–121.8].

**Fig. 2:**
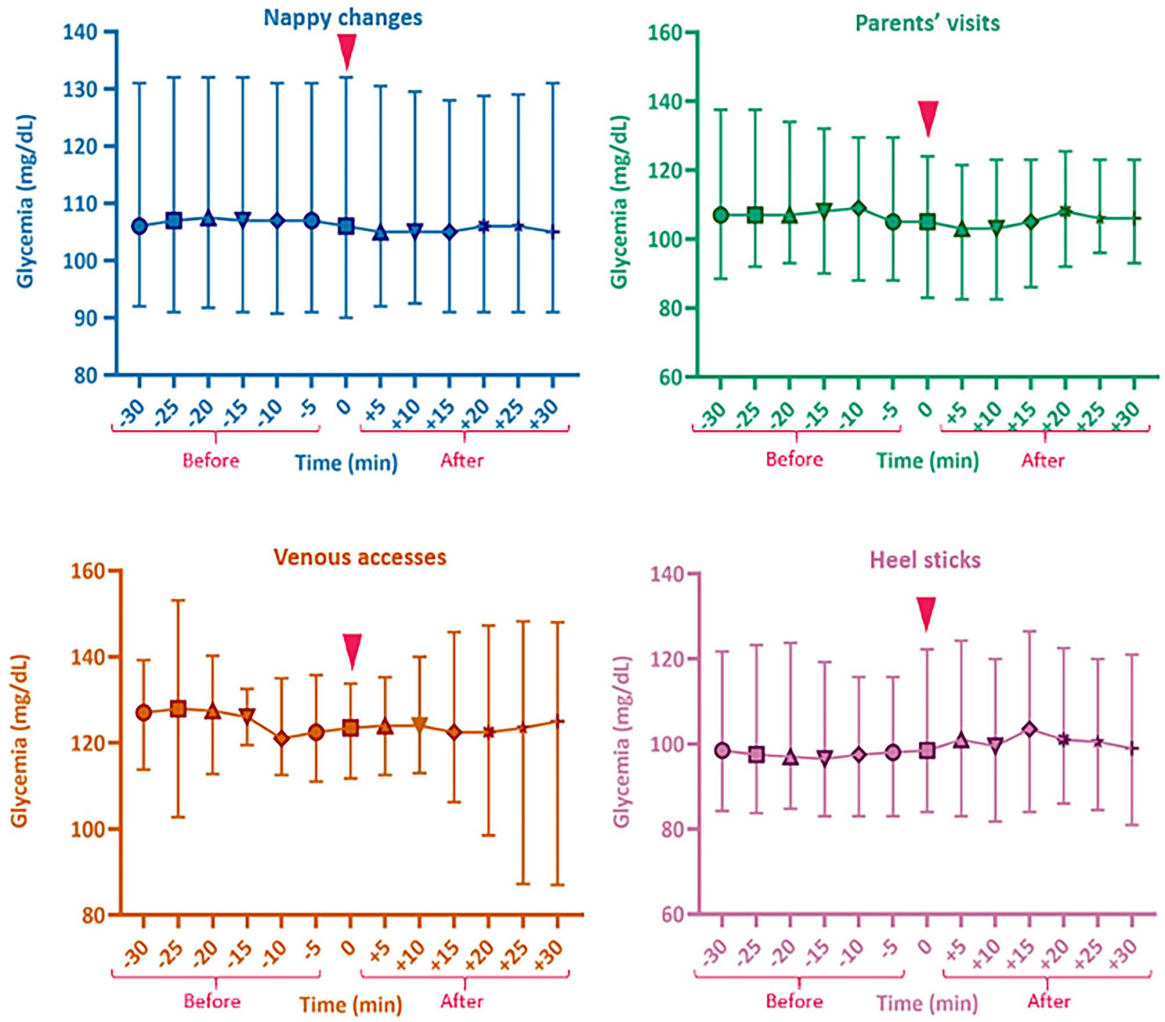
Sensor glucose profile before and after the procedures. Data are expressed as median (25th–75th centile)

Figure 2 displays the glycemic trends during the four procedures. Considering sensor glucose values 30 min before and after the nappy change, the median glucose values were respectively 107 mg/dL [IQR 91–131] and 105.5 mg/dL [IQR 91–130]; before parents’ visiting, the median was 107 mg/dL [IQR 89–132]; after that, it was 104.5 mg/dL [88.7–116.7]. The median of blood glucose values in the 30 min before the heel stick was 97 mg/dL [IQR 84–120]; after that, it was 101 mg/dL [IQR 84–122]. Before and after the introduction of a venous access, the median glucose was 125.5 mg/dL [IQR 112–137.2] and 124 mg/dL [IQR 101.7–143.]. There were no differences between the median sensor glucose values during the 30 min preceding and following the studied intervention. The mixed model analysis demonstrated the absence of a difference for the nappy change (*p* = 0.840), the parental presence (*p* > 0.99), and the venous access and the heel stick (*p* = 0.968 and *p* = 0.975, respectively), after adjusting for gestational age and birth weight.

The percentage glucose change from baseline did not differ across the four groups (*p* = 0.850) with a median change of −1.4% [−6.5, +6.6] for the nappy change, +2.8% [−3.4, +0.5] before and after parents’ arrival, −2.4% [−7.1, 4.2] for the venous access, and −1.6% [−8.3, 7.0] for the heel stick.

**Fig. 3:**
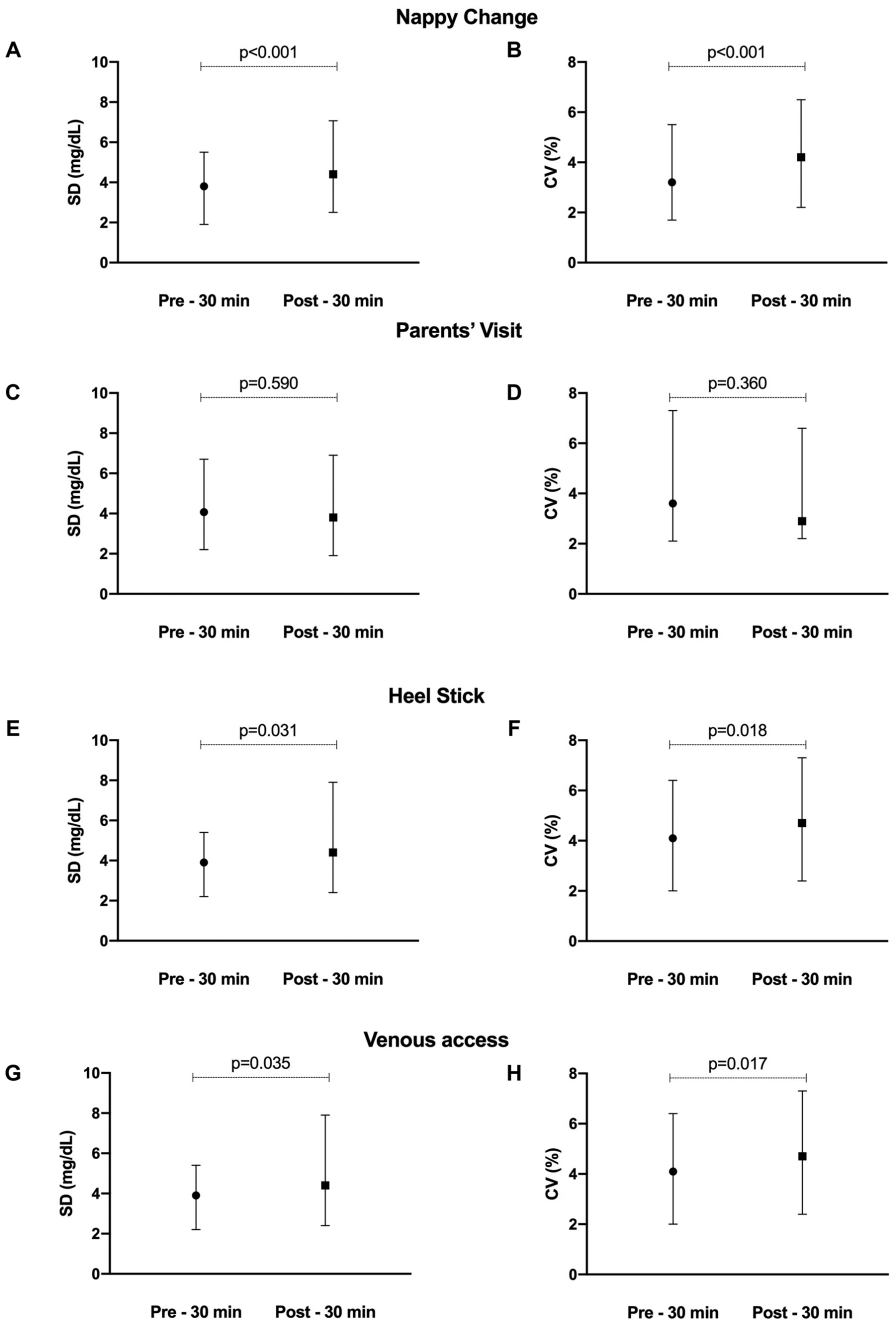
Glucose variability during the study procedures, expressed as CV and SD. Data are expressed as median (25th–75th centile). CV, coefficient of variation; SD, standard deviation

With respect to nappy change, SD was higher after the procedure (3.8 mg/dL [IQR 1.9–5.5] vs 4.4 mg/dL [IQR 2.5–7.07], *p* < 0.001) with a similar difference for the coefficient of variation (3.2% [IQR 1.7–5.5] vs 4.2% [IQR 2.2–6.5], *p* < 0.001) (Fig. 3A, B).

During parents’ visits, SD and CV did not change (4.07 mg/dL [IQR 2.23–6.72] vs 3.8 mg/dL [IQR 1.9–6.9], *p* = 0.59 for SD) (3.6% [IQR 2.1–7.3] vs 2.94% [IQR 2.2–6.6], *p* = 0.37 for CV) (Fig. 3C, D).

Conversely, glucose variability increased after heel stick procedures, with both SD and CV increase during the 30 min following the procedures {(3.9 mg/dL [IQR 2.2–5.4] vs 4.4 mg/dL [IQR 2.4–7.9], *p* = 0.0306 for SD) and (4.1% [IQR 2–6.4] vs 4.7% [IQR 2.4–7.3], *p* = 0.0178 for CV)} (Fig. 3E, F).

Glucose variability during venipuncture exhibited the same trend with a lower SD and CV before the procedure {(2.7 mg/dL [IQR 1.7–4.9] vs 6.9 mg/dL [IQR 3.7–10.4], *p* = 0.035 for the SD) and (2.14% [IQR 1.2–4.9] vs 4.56% [IQR 2.7–10.3], *p* = 0.017 for the CV)} (Fig. 3G, H).

Infants were monitored for signs of adverse events at sensor insertion sites, such as infection, irritation, subcutaneous hemorrhage, and subcutaneous sensor wire breakage, with no adverse events reported.

## Discussion

We demonstrated that non-invasive procedures, such as nappy change, increase glucose variability, as quantified during CGM use in preterm neonates, while parental presence is not associated with higher glucose variability or increase of glucose values.

Minimally invasive procedures such as nappy change have been previously associated with salivary cortisol fluctuations in preterm infants with a slower return to baseline of cortisol values after the procedure in preterm than full-term infants [3, 11].

Herein, we adopt a relatively new approach in neonatal care to quantify glucose fluctuations, based on CGM, which allows the quantification of glucose variability—and supposedly of the stress associated with such a glucose fluctuation—over a prolonged time period.

To this end, previous findings obtained by measuring salivary catecholamines increase after neonatal care procedures, like the diaper change, sheets’ change, and baby cleaning [12], cannot be reproduced on a larger temporal scale due to the nature of the measure and its costs, while CGM use could be a promising alternative to such a goal.

Conversely, the absence of glucose variability associated with parental presence underlies the key importance of promoting the early mother–infant relationship, even in NICU [13] and in the presence of institutional minimal handling practices for very preterm infants.

Heel stick and venipuncture are frequent procedures in NICU (> 2 times per day in the first week of birth [15]) and have been both associated with increased skin conductance—a stress marker in preterm—and the serum cortisol in preterm infants [14]. We confirmed such a finding with an increased glucose variability (SD and CV) after both procedures, in spite of the absence of significant glucose raise.

The overall sensor performance was featured by a limited loss of sensor signal and data—less than 2% of total time monitoring—confirming the reliability of such an instrument in this population observed by our and other groups [1–3].

The clinical relevance of an increased glucose variability has not been fully exploited in preterm infants. It has been associated with increased morbidity (late-onset sepsis) and mortality in the absence of CGM data [15].

In conclusion, for the first time using CGM, we demonstrated an increased glucose variability associated to non-invasive procedures in preterm infants, thus suggesting the need for enforcing minimal handling practice that could minimize neonate manipulation. Additionally, such a study suggests the use of CGM and glucose variability as a surrogate measure of discomfort in preterm neonates to be furtherly explored.

## Abbreviations

CGM: Continuous glucose monitoring;
CV: Coefficient of variation
SD: Standard deviation
